# The Relative Early Decrease in Platelet Count Is Associated With Mortality in Post-cardiotomy Patients Undergoing Venoarterial Extracorporeal Membrane Oxygenation

**DOI:** 10.3389/fmed.2021.733946

**Published:** 2021-11-04

**Authors:** Liangshan Wang, Juanjuan Shao, Chengcheng Shao, Hong Wang, Ming Jia, Xiaotong Hou

**Affiliations:** Center for Cardiac Intensive Care, Beijing Anzhen Hospital, Capital Medical University, Beijing, China

**Keywords:** post-cardiotomy cardiogenic shock, venoarterial extracorporeal membrane oxygenation, platelet count, mortality, decrease

## Abstract

**Background:** The relationship between the magnitude of platelet count decrease and mortality in post-cardiotomy cardiogenic shock (PCS) patients undergoing venoarterial extracorporeal membrane oxygenation (VA-ECMO) has not been well-reported. This study was designed to evaluate the association between the relative decrease in platelet count (RelΔplatelet) at day 1 from VA-ECMO initiation and in-hospital mortality in PCS patients.

**Methods:** Patients (*n* = 178) who received VA-ECMO for refractory PCS between January 2016 and December 2018 at the Beijing Anzhen Hospital were reviewed retrospectively. Multivariable logistic regression analyses were performed to assess the association between RelΔplatelet and in-hospital mortality.

**Results:** One hundred and sixteen patients (65%) were weaned from VA-ECMO, and 84 patients (47%) survived to hospital discharge. The median [interquartile range (IQR)] time on VA-ECMO support was 5 (3–6) days. The median (IQR) RelΔ platelet was 41% (26–59%). Patients with a RelΔ platelet ≥ 50% had an increased mortality compared to those with a RelΔ platelet < 50% (57 vs. 37%; *p* < 0.001). A large RelΔplatelet (≥50%) was independently associated with in-hospital mortality after controlling for potential confounders (OR 8.93; 95% CI 4.22–18.89; *p* < 0.001). The area under the receiver operating characteristic curve for RelΔ platelet was 0.78 (95% CI, 0.71–0.85), which was better than that of platelet count at day 1 (0.69; 95% CI, 0.61–0.77).

**Conclusions:** In patients receiving VA-ECMO for post-cardiotomy cardiogenic shock, a large relative decrease in platelet count in the first day after ECMO initiation is independently associated with an increased in-hospital mortality.

## Introduction

Venoarterial extracorporeal membrane oxygenation (VA-ECMO) has increasingly used as a rescue strategy to provide temporary circulatory and respiratory support allowing cardiac function recovery or bridging to additional therapeutic alternatives in patients with refractory post-cardiotomy cardiogenic shock (PCS) (1, 2). Despite major innovations in ECMO support over the last few decades, this rescue therapy is still marred by high rates of complications and mortality (3, 4). Particularly, patients requiring VA-ECMO are at increased risk of developing thrombopenia (5). Platelet activation, inflammatory and coagulative cascade activation, and consumption by the extracorporeal circuit play a major role in this context (6–8). Moreover, the use of unfractionated heparin during VA-ECMO may cause thrombocytopenia, which is an immune-mediated hypercoagulable disorder (9). Thrombocytopenia has been associated with increased mortality in patients undergoing VA-ECMO after cardiac surgery and has been incorporated in newer prognostic scoring systems (10). Previous studies investigating the prognostic significance of thrombocytopenia have focused on absolute platelet counts (11). However, limited data exist on the magnitude of platelet count decrease in PCS patients who are supported with VA-ECMO. In addition, the association between the magnitude of platelet count decrease and mortality in these patients has not been well-reported.

The primary objective of this single-center retrospective study was to evaluate whether the magnitude of platelet count decrease in the first day of ECMO initiation was associated with in-hospital mortality after controlling for potential confounders. Other clinical outcomes were also evaluated.

## Methods

### Patients

We retrospectively evaluated consecutive patients who received VA-ECMO from January 2016 and December 2018 at the Beijing Anzhen Hospital. Patients who received VA-ECMO for refractory PCS after cardiac surgery were included. The clinical criteria for PCS included the following (12): left atrial pressure >15 mmHg; central venous pressure >12 mmHg; metabolic acidosis (i.e., pH < 7.3 with serum lactate >3.0 mmol/L); end-organ hypoperfusion (urine output <30 mL/h); cardiac index <2.2 L/min/m^2^; and systolic blood pressure <80 mmHg despite adequate filling volumes, use of multiple adrenergic agents (epinephrine > 0.1 μg/kg/min or dobutamine > 10 μg/kg/min, norepinephrine > 0.1 μg/kg/min), or an intra-aortic balloon pump (IABP). Exclusion criteria for patient selection from our institutional ECMO database were an age <18 years, venovenous ECMO support for acute respiratory failure, ECMO initiation for non-PCS (e.g., fulminant myocarditis, myocardial infarction-associated cardiogenic shock), and ECMO initiation before cardiac surgery. The study was approved by the institutional ethics committee/review board of the Beijing Anzhen Hospital (2021020X), and the requirement for informed patient consent was waived in view of the retrospective nature of the study.

### ECMO Implantation and Management

The details regarding VA-ECMO initiation and management have been described previously (13). Briefly, all procedures were performed by trained ECMO team members. VA-ECMO support was initiated *via* peripheral cannulation through the femoral route with the semi-open method, and an additional 6 Fr catheter was systematically inserted distally into the femoral artery to prevent severe leg ischemia. ECMO blood flow was adjusted on based on clinical assessments (e.g., mixed venous oxygen saturation, evidence of hypoperfusion, resolution of hyperlactatemia, and normalization of mean arterial pressure). Intravenous unfractionated heparin was given to maintain an activated clotting time of 180–210 s, or an activated partial thromboplastin time of 1.5–2 times normal. ECMO-related complications were carefully monitored. ECMO weaning was performed in patients who fulfilled our published institutional weaning criteria and passed an ECMO weaning trial consisting in decreasing and clamping ECMO flow (14, 15). In general, the patient should have a pulsatile arterial waveform for at least 24 h; be hemodynamically stable, with baseline mean arterial pressure >60 mmHg with no or low doses of catecholamines; should have left ventricular ejection fraction (LVEF) of 35%, and an aortic velocity time integral (VTI) of ≥12 cm; and have recovered from major metabolic disturbances.

### Data Collection and Outcome Variables

The following information was recorded retrospectively: age; sex; weight; comorbid conditions; primary diagnosis; operative parameters; IABP use; ECMO peak flow; pre-ECMO left ventricular ejection fraction (LVEF); pre-ECMO cardiac arrest; platelet count before ECMO initiation; lowest platelet count at day 1 from ECMO initiation; peak serum lactate at day 1. Magnitude of platelet count decline was defined as the relative decrease in platelet count (RelΔ platelet), which was calculated from the following formula: RelΔ platelet = (platelet count at day 1- pre-ECMO platelet count)/(pre-ECMO platelet count).

The primary outcome was in-hospital mortality, defined as death from any cause occurring in patients who were treated by post-cardiotomy VA-ECMO. Secondary outcomes included length of intensive care unit (ICU) stay, length of hospital stay, ECMO duration, survival to ECMO weaning, continuous renal replacement therapy (CRRT), bleeding need thoracotomy, leg ischemia requiring fasciotomy, and major neurological complications (brain death, ischemic stroke, hemorrhagic stroke, and anoxic encephalopathy). Weaning was considered unsuccessful if ECMO re-cannulation was required within 2 days of decannulation.

### Statistical Analysis

All the analyses were performed with STATA/SE 12.0 (StataCorp, College Station, TX, USA) and SPSS 25.0 (IBM Corp, Armonk, NY, USA). The patients were grouped in two groups according to RelΔ platelet (<50, ≥50%; the optimal RelΔ platelet cut-off value for predicting mortality was 49.5%). The characteristics of patients were reported as proportions for categorical variables and as median [interquartile range (IQR)] for continuous variables. Categorical variables were compared with chi-square or Fisher's exact-tests, and continuous variables were compared with the Mann–Whitney *U*-test. We plotted the in-hospital mortality according to intervals of RelΔ platelet to evaluate the nature of the relationship between the two variables. The variables affecting the in-hospital mortality that were significant (*p* < 0.2) in univariable analysis were included in multivariable logistic regression with backward stepwise analysis. Discriminatory performance of RelΔ platelet vs. lowest platelet count at day 1 were compared using the area under the receiver operating characteristics curve (AUROC). Short-term survival was modeled using the Kaplan-Meier method, and inter-group comparisons were performed with the log-rank test. *P*-values < 0.05 were considered significant.

## Results

### Populations

**Figure 1:**
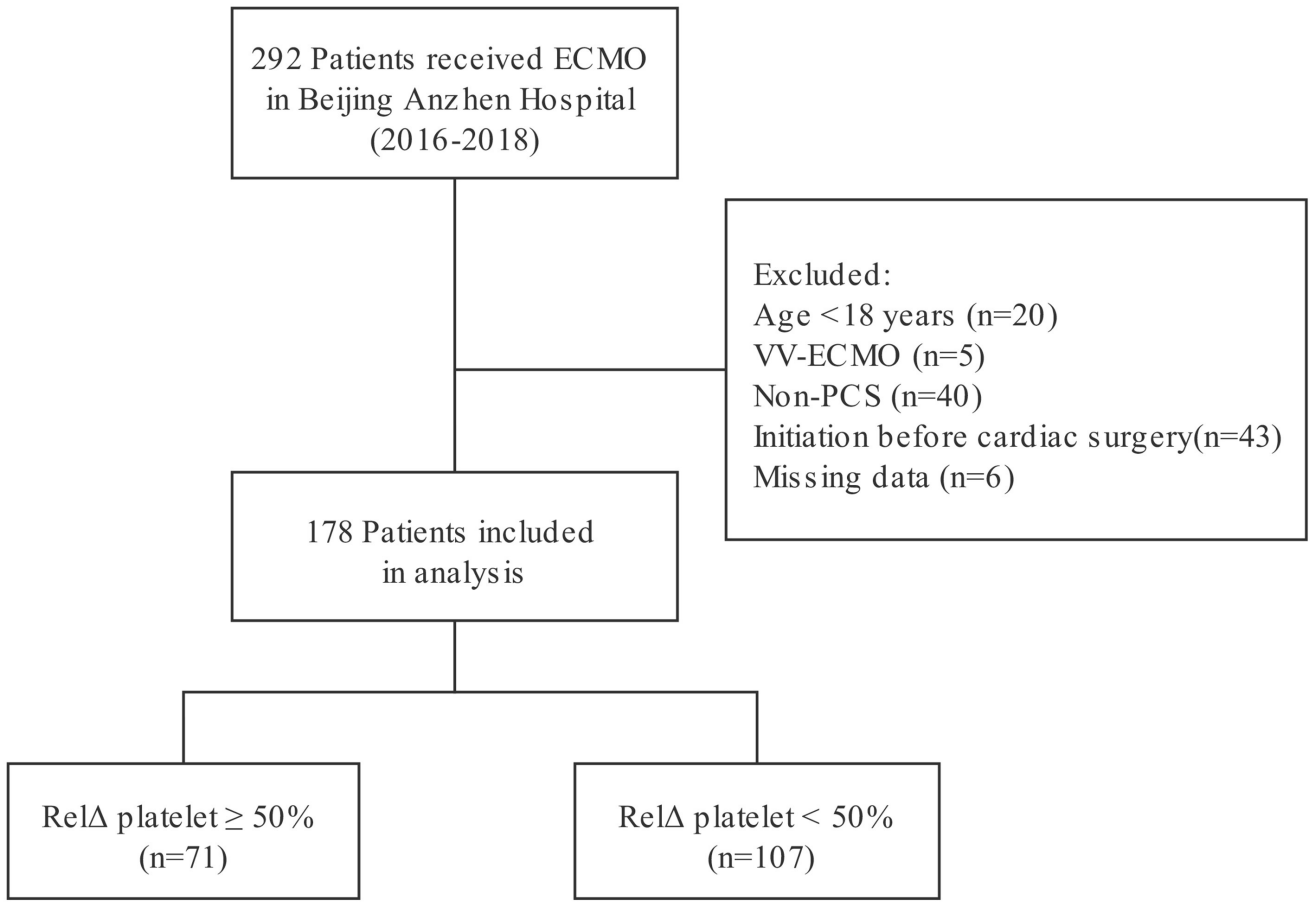
Flow diagram for selection of patients.

Two hundred and ninety-two patients received ECMO treatment over a 3-year period. Among those patients, 114 patients were excluded because of an age of <18 years (*n* = 20), acute respiratory failure treated with venovenous ECMO (*n* = 5), non-PCS supported with VA-ECMO (*n* = 40), ECMO initiation before cardiac surgery (*n* = 43), or unavailable medical records (*n* = 6). Finally, 178 PCS patients who were treated by VA-ECMO were included in analysis (Figure 1). Clinical characteristics of the patients according to RelΔ platelet are presented in the Table 1. The median (IQR) age of patients was 60 (52–66) years and 138 were male (78%). Eighty-seven patients (49%) were diagnosed with coronary heart disease, and 9 (5%) patients needed heart transplantation. Sixty-eight patients (38%) were not successfully weaned from cardiopulmonary bypass (CPB) due to PCS requiring transition to ECMO. One hundred and fifteen patients (65%) received additional IABP therapy, and median (IQR) pre-ECMO LVEF was 24% (17–30%). Fifty-four patients (30%) suffered from cardiac arrest before VA-ECMO implantation. The median (IQR) platelet count at day 1 was 61 (40–88) × 10^9^/L, and the median (IQR) serum lactate at day 1 was 13.0 (8.7–19.0) mmol/L. The median (IQR) RelΔ platelet was 41% (26–59%). There was no significant difference in clinical characteristics between patients with a RelΔ platelet ≥ 50% and patients with a RelΔ platelet < 50%, except for a lower platelet count in patients with a RelΔ platelet ≥ 50%.

**Table 1:** Clinical characteristics of the patients.

**Characteristic**	**Overall**	**RelΔ platelet ≥ 50%**	**RelΔ platelet < 50%**	***P-*** **value**
	**(***n*** = 178)**	**(***n*** = 71)**	**(***n*** = 107)**	
Age, years	60 (52–66)	61 (54–67)	60 (49–66)	0.330
Male	138 (78)	57 (80)	81 (76)	0.473
Weight, kg	70 (60–78)	70 (60–76)	70 (60–78)	0.644
**Comorbid conditions**				
Hypertension	99 (56)	41 (58)	58 (54)	0.642
Diabetes	41 (23)	16 (23)	25 (23)	0.898
Smoking	74 (42)	30 (42)	44 (41)	0.881
Recent MI	28 (16)	12 (17)	16 (15)	0.727
Stroke	6 (3)	2 (3)	4 (4)	0.736
Arrhythmia	18 (10)	10 (14)	8 (7)	0.152
**Diagnosis**				0.805
Coronary heart disease	87 (49)	38 (54)	49 (46)	
Valvular heart disease	36 (20)	13 (18)	23 (21)	
Aortic disease	17 (10)	7 (11)	10 (9)	
Coronary and valvular heart disease	22 (12)	6 (8)	16 (15)	
Heart transplantation	9 (5)	4 (6)	5 (5)	
Other	7 (4)	3 (4)	4 (4)	
**Operative parameters**				
Redo cardiac surgery	26 (15)	8 (11)	18 (17)	0.304
Emergency operation	17 (10)	7 (10)	10 (9)	0.909
Operative time, min	465 (300–600)	510 (300–665)	450 (300–555)	0.108
CPB time, min	151 (0–224)	159 (0–222)	137 (0–234)	0.882
Cross clamp time, min	55 (0–111)	33 (0–118)	58 (0–109)	0.715
Unsuccessful weaning off CPB	68 (38)	27 (38)	41 (38)	0.969
IABP use	115 (65)	49 (69)	66 (62)	0.317
ECMO peak flow, L/min	3.9 (3.4–4.0)	3.8 (3.5–4.0)	3.7 (3.4–3.9)	0.442
Pre-ECMO LVEF, %	24 (17–30)	24 (17–31)	23 (15–30)	0.911
Pre-ECMO cardiac arrest	54 (30)	23 (32)	31 (29)	0.627
Platelet count at day 1, × 10^9^/L^*^	61 (40–88)	45 (26–57)	72 (56–110)	<0.001
Serum lactate at day 1, mmol/L^*^	13.0 (8.7–19.0)	14.2 (9.7–19.6)	11.6 (8.1–19.0)	0.186

*Data are presented as medians (25th−75th percentile) or n (%)*.

*MI, myocardial infraction; CPB, cardiopulmonary bypass; IABP, intra-aortic balloon pump; ECMO, extracorporeal membrane oxygenation; LVEF, left ventricular ejection fraction; RelΔ platelet, relative decrease in platelet count*.

* *Worse value within 24 h after ECMO initiation*.

### Patient Outcomes

The hospital outcomes for all the study patients are listed in Table 2. One hundred and sixteen patients (65%) were weaned from VA-ECMO, and 84 patients (47%) survived to hospital discharge. The median (IQR) time on VA-ECMO support was 5 (3–6) days. The median (IQR) length of ICU stay and hospital stay duration were 8 (5–12) and 19 (12–26) days, respectively. Ninety-five (53%) patients required CRRT for renal failure. Twenty-nine (16%) patients underwent repeat thoracotomy for bleeding, and repeat thoracotomy was significantly more frequent in patients with a RelΔ platelet ≥ 50% as compared to patients with a RelΔ platelet < 50% (17 vs. 12%; *p* = 0.024). Seven patients (4%) required fasciotomy due to severe limb ischemia. Major neurologic complications were found in 31 (17%) of the patients and occurred more frequently in patients with a RelΔ platelet ≥ 50% as compared to patients with a RelΔ platelet < 50% (19 vs. 12%; *p* = 0.007).

**Table 2:** Outcomes.

**Outcome variables**	**Overall**	**RelΔ platelet ≥ 50%**	**RelΔ platelet < 50%**	***P-*** **value**
	**(***n*** = 178)**	**(***n*** = 71)**	**(***n*** = 107)**	
In-hospital mortality	94 (53)	57 (80)	37 (35)	<0.001
ECMO duration, days	5 (3–6)	5 (3–6)	5 (3–6)	0.395
Hospital stay, days	19 (12–26)	17 (11–25)	20 (14–27)	0.191
ICU stay, days	8 (5–12)	8 (5–13)	9 (6–12)	0.373
Successful weaning off ECMO	116 (65)	33 (46)	83 (78)	<0.001
CRRT	95 (53)	46 (65)	49 (46)	0.013
Bleeding need thoracotomy	29 (16)	17 (24)	12 (11)	0.024
Limb ischemia required fasciotomy	7 (4)	4 (6)	3 (3)	0.577
**Major neurological complications**	31 (17)	19 (6)	12 (22)	0.007
Brain death	6 (3)	6 (0)	0 (5)	0.008
Ischemic stroke	13 (7)	6 (4)	7 (8)	0.632
Hemorrhagic Stroke	6 (3)	3 (0)	3 (5)	0.607
Anoxic encephalopathy	6 (3)	4 (2)	2 (4)	0.348

*Data are presented as medians (25th−75th percentile) or n (%)*.

*ECMO, extracorporeal membrane oxygenation; ICU, intensive care unit; CRRT, continuous renal replacement therapy; RelΔ platelet, relative decrease in platelet count*.

### RelΔ Platelet and In-Hospital Mortality

**Figure 2:**
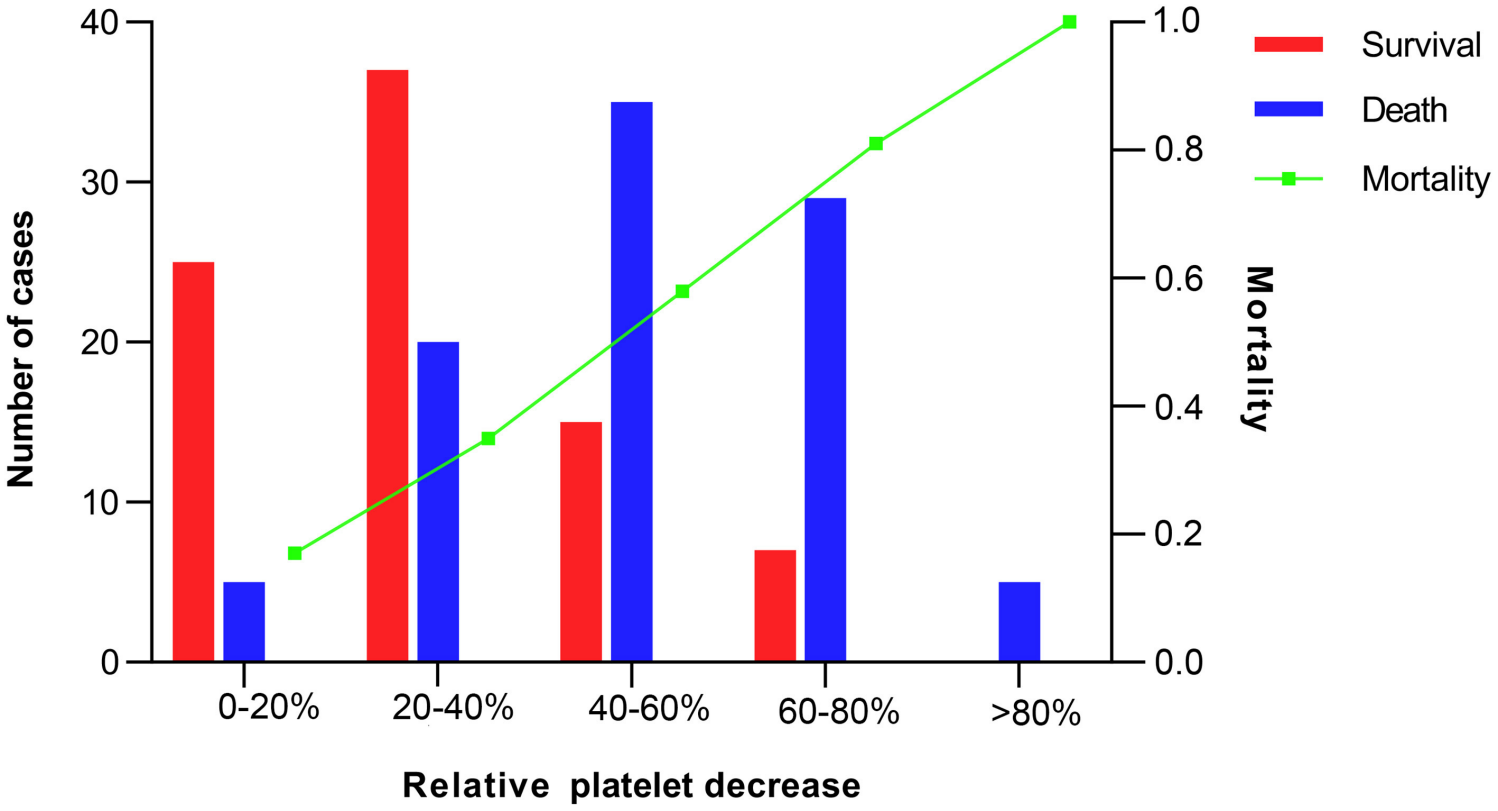
Number of cases and mortality according to intervals of relative decrease in platelet count.

**Figure 3:**
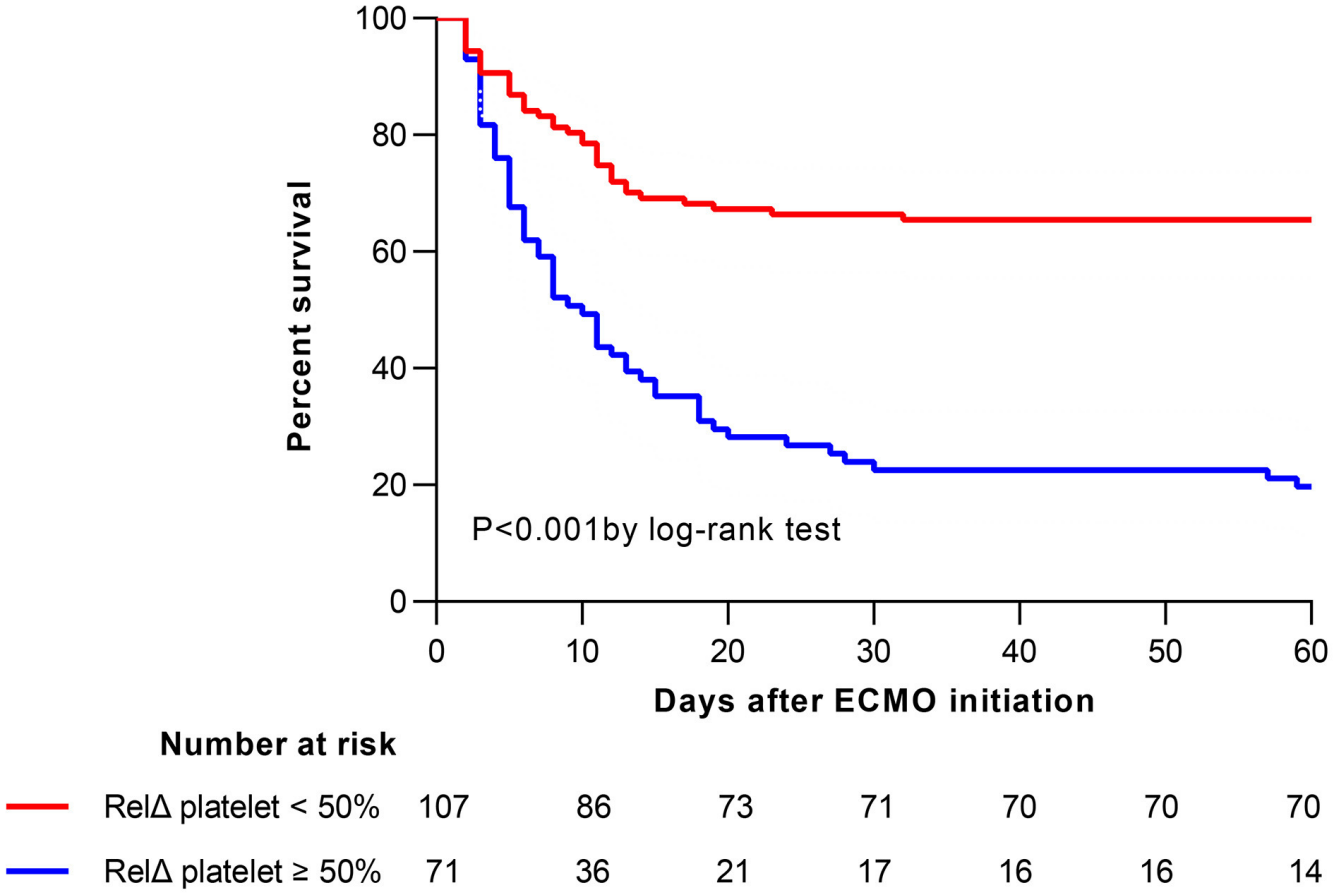
Kaplan-Meier estimates of cumulative probabilities of 60-day survival for patients according to RelΔ platelet ≥50 or <50%. ECMO, extracorporeal membrane oxygenation; RelΔ platelet, relative decrease in platelet count.

**Figure 4:**
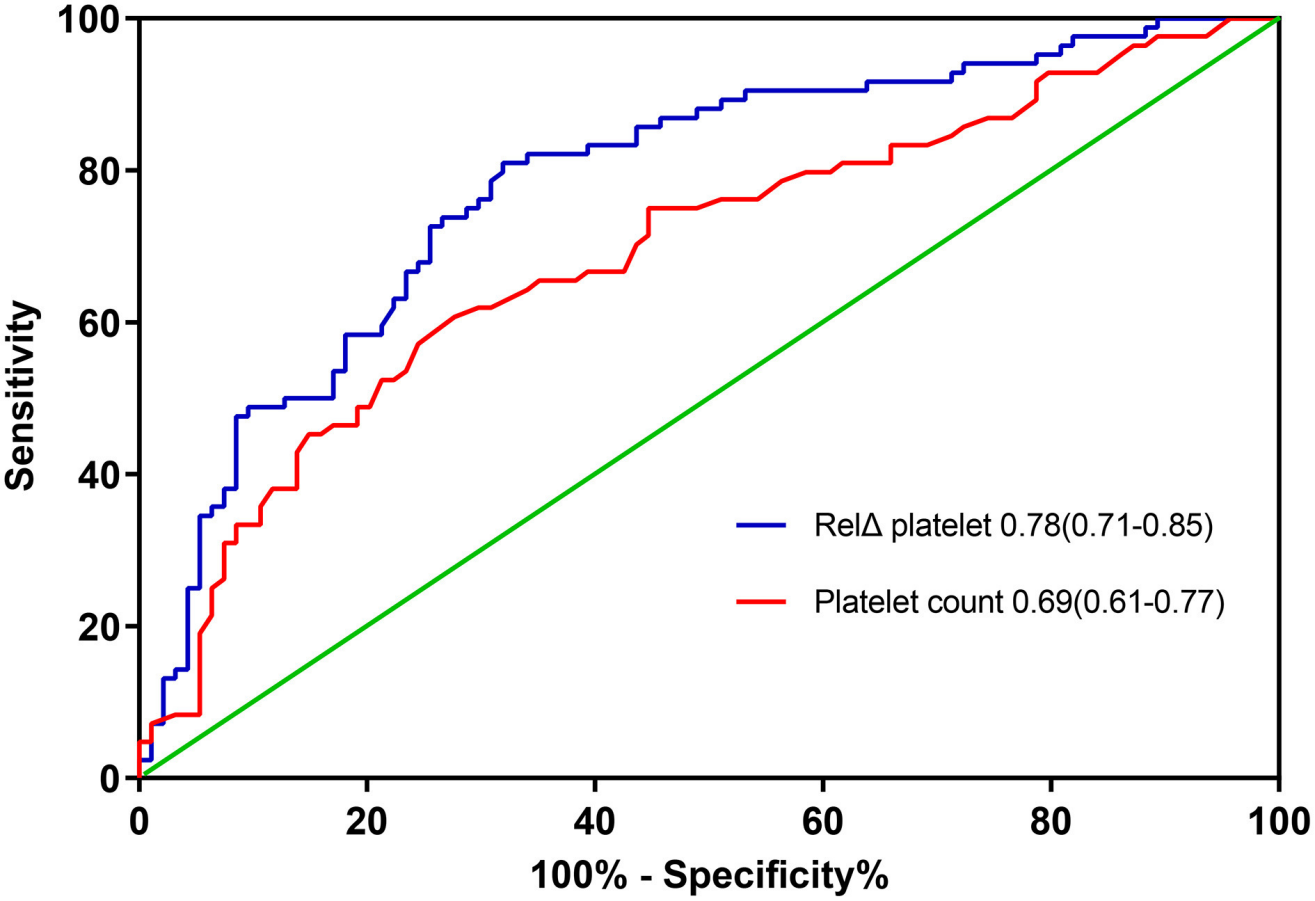
The areas under the receiver operating characteristic curves for predicting in-hospital death. RelΔ platelet, relative decrease in platelet count.

The overall in-hospital mortality was 53%. A higher RelΔ platelet was associated with a increased mortality, and only 3% of the patients had a RelΔ platelet > 80% (Figure 2). Patients with a RelΔ platelet ≥ 50% had a significantly increased mortality compared to patients with a RelΔ platelet < 50% (57 vs. 37%; *p* < 0.001 by log-rank-test; Figure 3). In multivariable logistic regression analyses, with adjustment for sex and serum lactate, RelΔ platelet ≥ 50% was independently associated with in-hospital mortality (OR 8.93; 95% CI 4.22–18.89; *p* < 0.001; Table 3). The AUROC for RelΔ platelet was 0.78 (95% CI, 0.71–0.85; Figure 4), which was better than that of platelet count (0.69; 95% CI, 0.61–0.77).

**Table 3:** Logistic regression analyses for in-hospital mortality.

	**Univariable**		**Multivariable**	
	**analysis**		**analysis**	
**Parameter**	**OR (95%CI)**	***P***	**OR (95%CI)**	***P***
Age (+10 years)	1.43 (1.10–1.86)	0.008		
Female	2.20 (1.05–4.62)	0.037	3.42 (1.46–8.01)	0.005
Diabetes	1.76 (0.86–3.61)	0.123		
Heart transplantation	0.24 (0.05–1.19)	0.080		
Redo cardiac surgery	0.51 (0.22–1.19)	0.117		
Serum lactate	1.07 (1.01–1.12)	0.016	1.07 (1.01–1.13)	0.016
RelΔplatelet (+10%)	1.82 (1.50–2.22)	<0.001		
**RelΔplatelet ≥ 50%**	7.70 (3.80–15.63)	<0.001	8.93 (4.22–18.89)	<0.001

*Variables with P < 0.2 by univariate analysis were subjected to multivariate analysis. The multivariate logistic regression analysis was set with entry and removal P-values of 0.05 and 0.1, respectively. RelΔ platelet, relative decrease in platelet count*.

## Discussion

In this cohort study of 178 patients who received VA-ECMO for PCS, we found that RelΔ platelet in the first day after ECMO initiation is independently associated with an increased in-hospital mortality, independent of sex and peak serum lactate at day 1. In addition, RelΔ platelet exhibited good performance as compared to absolute platelet count at day 1.

Despite increasing improvement in VA-ECMO technology and knowledge, thrombocytopenia is common in patients undergoing VA-ECMO, among whom more than 20% have platelet counts lower than 150 × 10^9^/L at some points during VA-ECMO (8). The initiation of ECMO is associated with an immediate and complex inflammatory reaction, similar to that seen in systemic inflammatory response syndrome (16). Moreover, the contact of blood with the surfaces of the extracorporeal circuit causes platelets activation and release of coagulation factors, an activation of the complement system. Thrombocytopenia might occur following cardiac surgery and ECMO due to extensive cross-talk between inflammation and coagulation, bleeding, consumption by the extracorporeal circuit, and oxidizing stress caused by high oxygen tension (17). It had been demonstrated that thrombocytopenia was related to longer ICU stays, a higher incidence of bleeding events, and higher mortality in ICU patients (18). However, there is a paucity of data on patients receiving VA-ECMO for PCS. In a recent retrospective study including 300 patients with left ventricular dysfunction after cardiac surgery (11), moderate (<100–50 × 10^9^/L), severe (49–20 × 10^9^/L), and very severe (<20 × 10^9^/L) were independently associated with 90-day mortality. In addition, the authors found that platelet count had a biphasic temporal pattern with an initial decrease until day 4–5 after the initiation of VA-ECMO.

Thrombocytopenia and a decrease in platelet count may reflect the same pathophysiologic disturbances, including disseminated intravascular coagulation, macrophage activation, sepsis, drug-induced toxicity, and unidentified factors (18). Although most studies of the prognostic impact of platelet counts focused on outcomes in pre-specified groups according to the severity of thrombocytopenia, changes in platelet counts may carry greater prognostic significance than absolute counts in several critical conditions (19). However, few studies evaluated the potential prognostic significance of declining platelet counts in patients supported with VA-ECMO after cardiac surgery. In our study, RelΔ platelet or a large RelΔ platelet (≥50%) in the first day after ECMO initiation is independently associated with in-hospital mortality, independent of sex and peak serum lactate at day 1. Importantly, RelΔ platelet had better discrimination than platelet count at day 1 in our cohort. As expected, severe bleeding events was more common in patients with a large RelΔ platelet ≥ 50%. Our study confirmed that the early decrease in platelet count had better prognostic significance than absolute counts in patients receiving VA-ECMO for PCS.

Platelet count has been incorporated in several prognostic scoring systems (10, 20). In the REMEMBER score, lowest platelet count <100 × 10^9^/L was associated with in-hospital mortality. Nevertheless, absolute platelet count cannot reflect changes in clinical status in patients whose platelet counts decrease but remained within the normal range. This might account for our findings that RelΔ platelet had better prognostic significance than platelet count. Thus, RelΔ platelet might help improve discrimination of new prognostic scoring systems for patients supported with VA-ECMO for PCS. Furthermore, RelΔ platelet at day 1 can be easily computed in real-time at the bedside and may afford clinicians the opportunity to evaluate the severity of illness before the development of other end-organ dysfunction, which may prompt earlier intervention, or a change in management strategy.

Our study has several limitations. First, it was a single-center, retrospective study which may limit the generalizability of our results. Second, bleeding and platelet transfusion would result in changes in platelet count. These factors might have affected the effect of RelΔ platelet on mortality.

Third, because left ventricular assist devices were not registered in China, no patients underwent ventricular assist device after VA-ECMO. The usefulness of VA-ECMO for PCS patients might have therefore been underestimated. Fourth, variables regarding platelet function, including platelet aggregation and activation of platelet, were not available. Finally, additional potential confounders undoubtedly exist, but it is unlikely the large magnitude of effect of RelΔ platelet has on mortality can all be explained by a yet undetermined variable. In the present study, we performed adjusted analyses to control for confounders in the evaluation of patients' outcomes.

## Conclusions

In patients receiving VA-ECMO for post-cardiotomy cardiogenic shock, a large relative decrease in platelet count in the first day after ECMO initiation is independently associated with an increased in-hospital mortality, independent of sex and peak serum lactate at day 1. Our study suggests that clinicians should monitor RelΔ platelet frequently and might identify high-risk patients early. Prospective studies are needed to externally validate the prognostic significance of RelΔ platelet in other populations of patients who received VA-ECMO for PCS.

## Data Availability Statement

The raw data supporting the conclusions of this article will be made available by the authors, without undue reservation.

## Ethics Statement

The studies involving human participants were reviewed and approved by Institutional Ethics Committee/Review Board of the Beijing Anzhen Hospital. The Ethics Committee waived the requirement of written informed consent for participation.

## Author Contributions

LW and XH: conception and design. LW, JS, CS, and MJ: analysis and interpretation. JS and LW: drafting of the manuscript. HW and XH: revision of the manuscript. All authors read and approved the final version.

## Funding

This work was supported by the National Key Research and Development Program of China (2016YFC1301001, to XH), Beijing Hospitals Authority Clinical Medicine Development of Special Funding Support (Code: ZYLX202111), and Beijing Hospitals Authority “Ascent Plan” (Code: FDL20190601).

## Conflict of Interest

The authors declare that the research was conducted in the absence of any commercial or financial relationships that could be construed as a potential conflict of interest.

## Publisher's Note

All claims expressed in this article are solely those of the authors and do not necessarily represent those of their affiliated organizations, or those of the publisher, the editors and the reviewers. Any product that may be evaluated in this article, or claim that may be made by its manufacturer, is not guaranteed or endorsed by the publisher.
